# Supplementary figures and images for: A Phenome-Wide Association Study of genes associated with COVID-19 severity reveals shared genetics with complex diseases in the Million Veteran Program
Source: PLoS Genet. 2022 Apr 28;18(4):e1010113. doi: 10.1371/journal.pgen.1010113 (PMC9049369; doi:10.1371/journal.pgen.1010113)

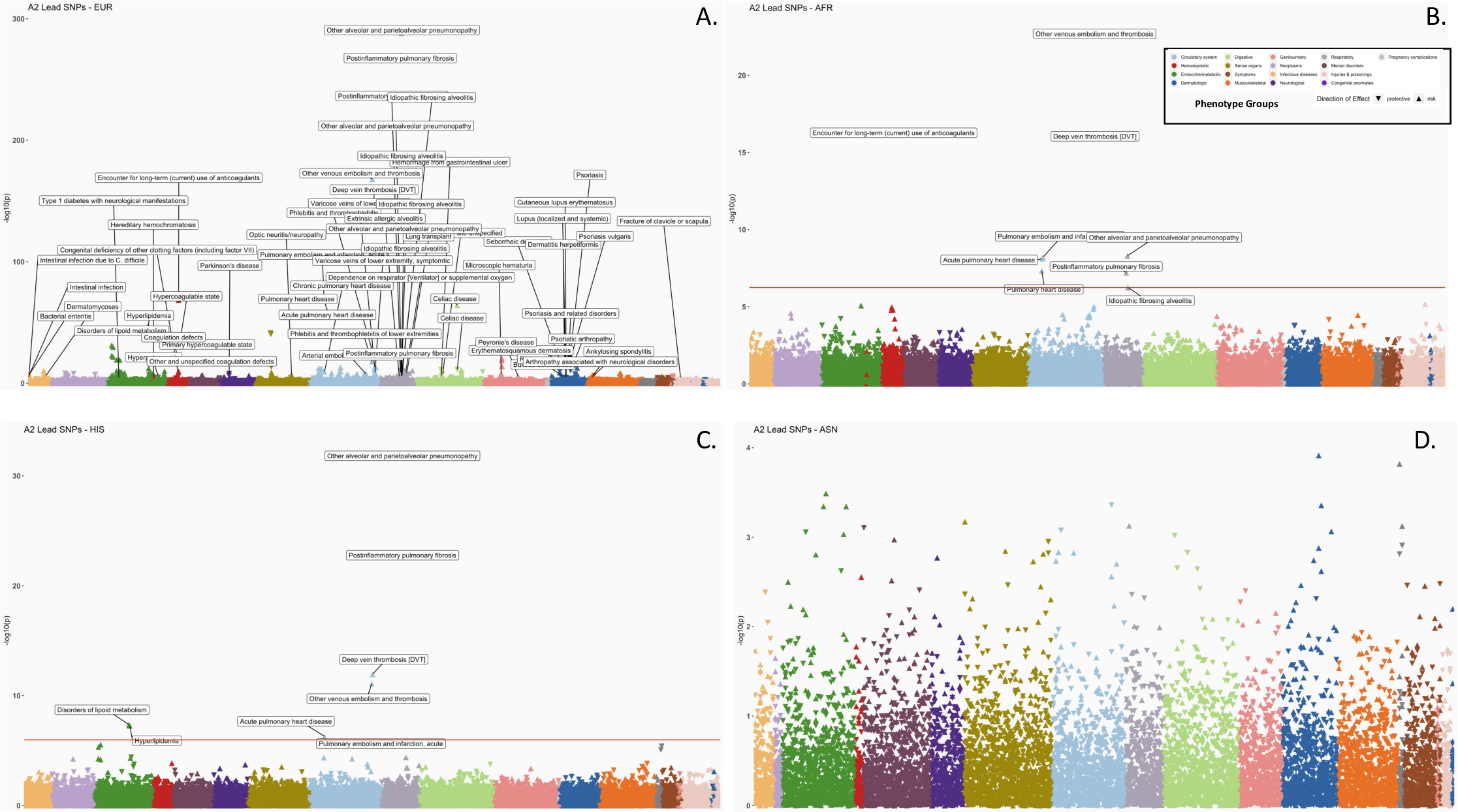

Supplement: S1 Fig — The PheWAS results of 48 SNPs from critical ill COVID GWAS by each ancestry a) European ancestry, b) African ancestry, c) Hispanic ancestry, and d) Asian ancestry. (TIF) [file pgen.1010113.s010.tif]

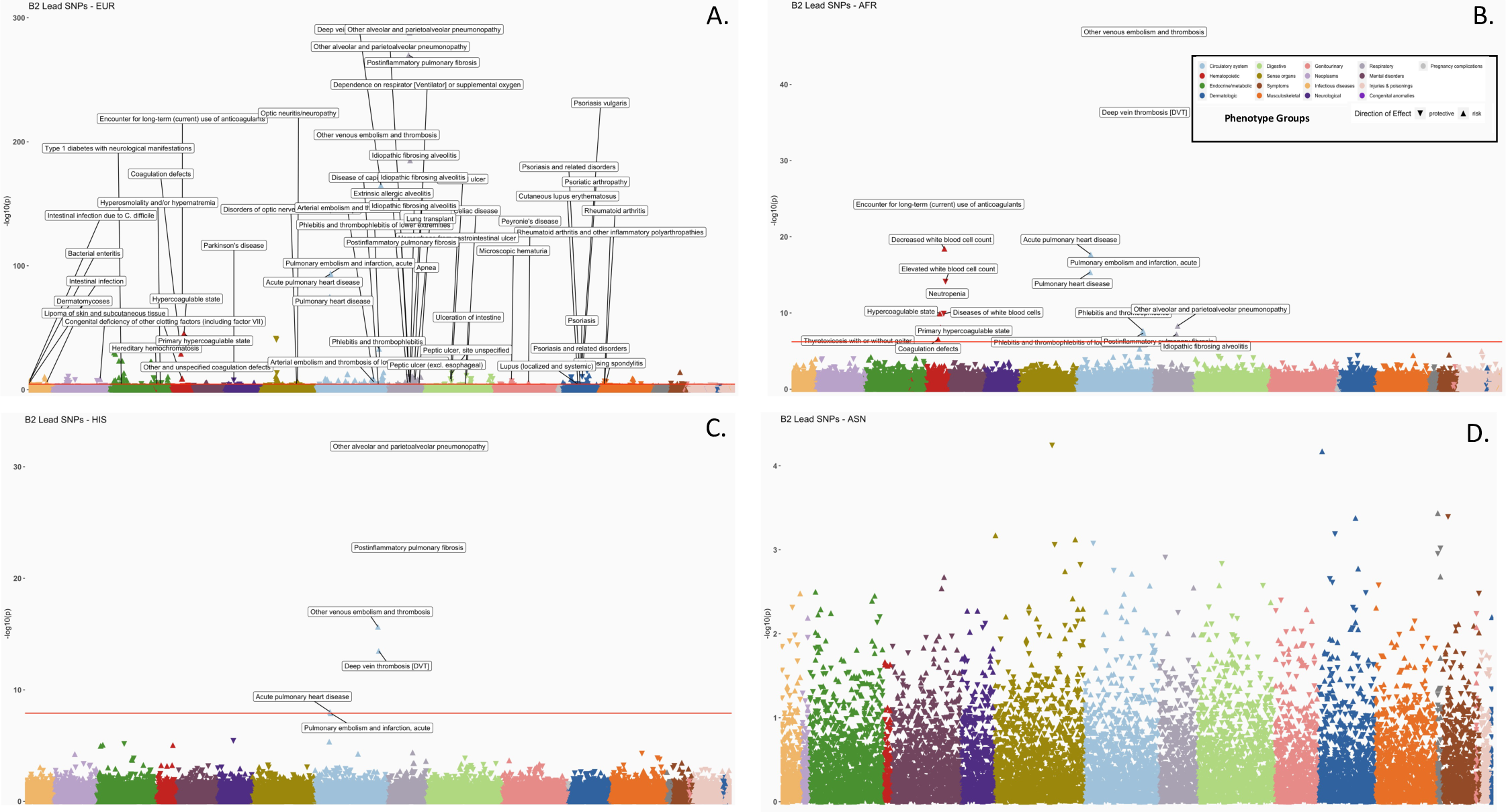

Supplement: S2 Fig — The PheWAS results of 39 SNPs from hospitalized COVID GWAS by each ancestry a) European ancestry, b) African ancestry, c) Hispanic ancestry, and d) Asian ancestry. (TIF) [file pgen.1010113.s011.tif]
