# Supplementary material for: A Phenome-Wide Association Study of genes associated with COVID-19 severity reveals shared genetics with complex diseases in the Million Veteran Program
Source: PLoS Genet. 2022 Apr 28;18(4):e1010113. doi: 10.1371/journal.pgen.1010113 (PMC9049369; doi:10.1371/journal.pgen.1010113)
Supplement: S1 Text — (DOCX) [file pgen.1010113.s012.docx]

**Supplemental Materials**

**A Phenome-Wide Association Study of genes associated with COVID-19 severity reveals shared genetics with complex diseases in the Million Veteran Program**

**VA Million Veteran Program COVID-19 Science Initiative Membership & Acknowledgements**

**VA Million Veteran Program COVID-19 Science Initiative**

**MVP COVID-19 Science Program Steering Committee**

- J. Michael Gaziano, M.D., M.P.H. (Co-Chair)
- Philip S. Tsao, Ph.D. (Co-Chair)
- Sumitra Muralidhar, Ph.D.
- Jean Beckham, Ph.D.
- Kyong-Mi Chang, M.D.
- Juan P. Casas, M.D., Ph.D.
- Kelly Cho, M.P.H., Ph.D.
- Saiju Pyarajan, Ph.D.
- Jennifer Huffman, Ph.D.
- Jennifer Moser, Ph.D.

**MVP COVID-19 Science Program Steering Committee Support**

- Lauren Thomann, M.P.H. (P&P Committee Representative, Working Group Coordinator)
- Helene Garcon, M.D. (Program Coordinator, Working Group Coordinator)
- Nicole Kosik, M.P.H. (Working Group Coordinator)

**OVID-19 Science Program Working Groups and Associated Chairs**

- COVID-19 Related PheWAS
  - Katherine Liao, M.D.
  - Scott Damrauer, M.D.
- Disease Mechanisms
  - Richard Hauger, M.D.
  - Shiuh-Wen Luoh, M.D., Ph.D.
  - Sudha Iyengar, Ph.D.
- Druggable Genome
  - Juan P. Casas, M.D., Ph.D.
- Genomics for Risk Prediction, PRS, and MR
  - Themistocles Assimes, M.D., Ph.D.
  - Panagiotis Roussos, M.D., Ph.D.
  - Robert Striker, M.D., Ph.D.
- GWAS & Downstream Analysis
  - Jennifer Huffman, Ph.D.
  - Yan Sun, Ph.D.
- Pharmacogenomics
  - Adriana Hung, M.D., M.P.H.
  - Sony Tuteja, Pharm.D., M.S.
- VA COVID-19 Shared Data Resource – Scott L. DuVall, Ph.D.; Kristine E. Lynch, Ph.D.; Elise Gatsby, M.P.H.

VA Informatics and Computing Infrastructure (VINCI), VA Salt Lake City Health Care System

- MVP COVID-19 Data Core – Kelly Cho, M.P.H., Ph.D.; Lauren Costa, M.P.H.; Anne Yuk-Lam Ho, M.P.H.; Rebecca Song, M.P.H.
