# Supplementary material for: A Phenome-Wide Association Study of genes associated with COVID-19 severity reveals shared genetics with complex diseases in the Million Veteran Program
Source: PLoS Genet. 2022 Apr 28;18(4):e1010113. doi: 10.1371/journal.pgen.1010113 (PMC9049369; doi:10.1371/journal.pgen.1010113)
Supplement: Attachment — Submitted filename: COVID_coverlett_PLoSGen_forReviewer.docx [file pgen.1010113.s013.docx]

October 15, 2021

Dear Reviewers:

We appreciate your taking the time to review our manuscript in consideration for publication in PLoS Genetics. The manuscript was originally submitted to medRxiv in March 2021 (<https://www.medrxiv.org/content/10.1101/2021.05.18.21257396v1>) and was peer-reviewed in other journals. Due to the fast pace of COVID-19 research, similar results were published by another group while our manuscript was under review. As a result, our prior Reviewers expressed concerns over whether our findings represented an advance in scientific knowledge relative to published data.

Since March, we updated and re-analyzed data from the COVID-19 Host Genetic Initiative (HGI) using the new release data (freeze 6, July 2021), which includes newly identified variants associated with severe COVID-19 phenotypes. As well, the PheWAS using this larger set of variants from HGI were performed on 658,582 Million Veteran Program (MVP) participants, an increase from our previous cohort of 455,683. The increased cohort size also represented a ~40K increase among participants of African ancestry, underscoring a strength of MVP as one of the most diverse biobanks in the world. These new data increased our power to detect associations in the overall cohort, as well as differences in associations across ancestries. The latter enabled us to perform more robust ancestry specific PheWAS analyses, addressing another major concern from prior reviewers.

Novel findings from our updated analyses include:

- A variant associated with severe COVID-19 was also associated with neutropenia among Veterans of African but not European ancestry providing intriguing data to inform heterogenous outcomes from SARS-CoV-2 infection
- Our updated analysis adds to the growing list of genetic variants shared between COVID-19 with autoimmune and inflammatory conditions where the direction of association with the variant are in opposite directions

We look forward to sharing the above and other findings from our comprehensive phenomics screen of COVID-19 variants across a broad range of human conditions, and to your reviews.

Thank you.

Sincerely yours,

Katherine P. Liao, MD, MPH

Associate Professor of Medicine and Biomedical Informatics, Harvard Medical School

Co-Director, Applied Bioinformatics Core, Massachusetts Veterans Epidemiology and Information Center, VA Boston Healthcare System

Associate Physician, Division of Rheumatology, Inflammation, and Immunity, Brigham and Women’s Hospital
