# Supplementary material for: A Phenome-Wide Association Study of genes associated with COVID-19 severity reveals shared genetics with complex diseases in the Million Veteran Program
Source: PLoS Genet. 2022 Apr 28;18(4):e1010113. doi: 10.1371/journal.pgen.1010113 (PMC9049369; doi:10.1371/journal.pgen.1010113)
Supplement: Attachment — Submitted filename: 2022.01.24_point-by-point_reviewer_response.docx [file pgen.1010113.s014.docx]

We would like to thank the Editors and Reviewers for their thoughtful comments which have helped us to improve our manuscript. Please see our point-by-point responses to comments below.

**Reviewer #1:**

1. I was involved in the review of this work when it was submitted to PLoS Medicine. I was invited to review the present manuscript as a revision to that submission. Overall, the paper is a substantial improvement over the previous version and addresses the majority of my earlier concerns. In particular, there is a much stronger justification for the choice of parameter values, and the removal of UKBB greatly improves the clarity of presentation. I only have one relatively minor concern remaining.

RESPONSE: We appreciate the Reviewer’s reassessment of our manuscript and providing positive feedback for the major updates since the last submission.

2. While it is common to adjust for the first k principal components (PCs) as a way to correct for broad-scale population structure, it may be good to show that the first 20 PCs do NOT align with the variables being modeled (associations with phecodes in this case). Otherwise, there is a concern that adjusting for PCs may lower the signal-to-noise ratio and artificially deflate the importance of one or more of the phecodes being investigated. The artificial deflation of importance may, for example, explain why the authors did not detect some of the known risk factors, such as COPD (Lines 268, 320).

RESPONSE: We agree with the Reviewer’s comment and there was internal debate regarding the number of PCs to use. A major issue was that the PheWAS entails evaluating associations between a SNP with over a thousand phenotypes across 4 racial/ethnic groups. The traditional approaches, e.g. the selection of number of PCs to include as covariates could be estimated from the genomic inflation factor (λ_GC_) for a given phenotype [1,2]. However, in this PheWAS we are testing only lead SNPs and thus was challenging to accurately compute the λ_GC_ . Moreover, due to the design of the PheWAS, where PheWAS was performed in each subpopulation- African, Asian, European and Hispanic, we chose a conservative approach to adjust the models using a standardized approach using 20 PCs for all populations and all Phecodes. This approach performed in previously published studies also facilitates comparison.^1^

We have added this important point to the limitations on page 17, line 560:

“To enable a transethnic study, we applied a conservative approach using 20 PCs to adjust all models, used in prior studies. One potential pitfall of this approach is that the models may be overadjusted and thus are more likely to miss few significant associations.”

3. Minor comments:

- eTable 1 -> S1 Table.
  RESPONSE: We have fixed the typo.
- The ancestry group acronyms EUR, AFR, etc. are used before they are defined.
  RESPONSE: We thank the Reviewer for this important point and have added a section, “Classification of race/ethnicity in MVP,” which now defines the acronyms. This update was also made in response to Reviewer # 2’s comment. Please see the updated section on page 7:

“Race and ethnicity was determined using the harmonized ancestry and race/ethnicity (HARE) method for four major groups^1^ corresponding to: (1) African ancestry, non-Hispanic Black (AFR), (2) Asian ancestry, non-Hispanic Asian (ASN), (3) European ancestry, non-Hispanic White (EUR), and (4) Hispanic ancestry (HIS). Individuals without a HARE classification were most likely be from ancestries with insufficient numbers to train the HARE algorithm including Native American, Alaska Native, and Pacific Islanders. Briefly, the HARE method was an algorithm developed to assign each subject to one of four groups using both self-reported race/ethnicity as the input to train a machine learning algorithm using ancestry informative genetic markers. This approach provides a classification for each individual leveraging information from genomic markers if self-reported race/ethnicity was missing, enabling analyses that can stratify by the four major groups. When comparing HARE vs self-reported race and ethnicity, the statistical error rate was estimated at 0.05-0.46%.”

- Line 237: “both severe and hospitalized COVID” -> “both critical and hospitalized COVID”
  RESPONSE: We have revised this sentence to read (now line number 279):

“Six variants were common to both critical and hospitalized COVID and had significant PheWAS associations, namely, variations nearest to the genes *ABO* (rs495828 and rs505922), *DPP9* (rs2277732), *MUC5B* (rs35705950), TYK2 (rs11085727), and *CCHCR1* (rs9501257)”

- Line 253: “have been reported as risk factors for or are complications associated with COVID-19 severity and mortality” -should cite the work being referenced in the sentence
  RESPONSE: We now included the relevant papers including:

1. CDC. About COVID-19 - CDC. : <https://www.cdc.gov/coronavirus/2019-ncov/cdcresponse/about-COVID-19.html>.
2. The GenOMICC Investigators et al. Genetic mechanisms of critical illness in COVID-19. Nature 591, 92–98 (2021).
3. Arentz, M. et al. Characteristics and Outcomes of 21 Critically Ill Patients With COVID-19 in Washington State. JAMA (2020) doi:10.1001/jama.2020.4326.
4. Gandhi, R. T., Lynch, J. B. & del Rio, C. Mild or Moderate Covid-19. N. Engl. J. Med. 383, 1757–1766 (2020).

- Figures 2A&C: Some of the labels are obscured by the overlap.
  RESPONSE: We appreciate this feedback from the Reviewer. Please see revised figures.
- Line 314: The acronym T2D (Type 2 Diabetes) has not been defined.
  RESPONSE: We have replaced T2D with Type 2 Diabetes in the text.
- Line 332: The sentence “The clinical data used in this study pre-dates the emergence of COVID-19…” feels out of place and repeats what was stated in the Methods. Consider cutting.
  RESPONSE: We appreciate the Reviewer’s suggestion and have removed that line.

**Reviewer #2:**

This manuscript reports a carefully executed and clearly described phenome wide association study (PheWAS) of SNPs associated with critical and hospitalized COVID in over 650K ancestrally diverse participants in the Million Veteran Program (MVP). The Methods are in general well defined, the Results clearly presented, and the Discussion clear, though in places a bit verbose.

1. Given that its ethnic and ancestral diversity is a major strength of the MVP, it is surprising that the authors reported their findings by genetic ancestry groups alone and not by self-identified race/ethnic groups as well. While the two are highly correlated, they are not identical, and at minimum a description of the correlation between the two should be provided. Preferable would be to repeat the findings stratifying by self-identified groups and describing observed similarities and differences. MVP is one of the very few cohorts that can do this; this opportunity should not be missed. They should also describe how individuals who did not fit into one of the four HARE groups were analyzed.

RESPONSE: We thank the Reviewer for this comment and the importance of highlighting the methods used to classify race and ethnicity in the MVP population. Ancestries in MVP were classified using a published method, the harmonized ancestry and race/ethnicity (HARE) algorithm^1^. This approach, draws information from both self-reported race/ethnicity and from ancestry informative markers, enabling classification of a large multi-ethnic cohort into non-overlapping populations. HARE is the standard for classifying race and ethnicity for genomic studies in MVP. When comparing HARE vs self-reported race and ethnicity, the statistical error rate was estimated to be 0.05-0.46%. We hope this explanation clarifies why the analyses were not also stratified by self-reported race/ethnicity.

An additional section in the Methods, “ Classification of race/ethnicity in MVP” to address this concern on page 7, line 199”

Race and ethnicity was determined using the harmonized ancestry and race/ethnicity (HARE) method for four major groups^1^ corresponding to: (1) African ancestry, non-Hispanic Black (AFR), (2) Asian ancestry, non-Hispanic Asian (AS), (3) European ancestry, (4) non-Hispanic White (EUR), and Hispanic ancestry (HIS). Individuals without a HARE classification were most likely be from ancestries with insufficient numbers to train the HARE algorithm including Native American, Alaska Native, and Pacific Islanders. Briefly, the HARE method was an algorithm developed to assign each subject to one of four groups using both self-reported race/ethnicity as the input to train a machine learning algorithm using ancestry informative genetic markers. This approach provides a classification for each individual leveraging information from genomic markers if self-reported race/ethnicity was missing, enabling analyses that can stratify by the four major groups. When comparing HARE vs self-reported race and ethnicity, the statistical error rate was estimated at 0.05-0.46%.

2. The Methods do not describe how SNPs were assigned to genes (or vice versa). Typically 85-90% of GWAS associations lie in intergenic regions, some of which are known to play important regulatory roles in other genes. The only reference I could find was in line 238, simply, “…variations nearest to the genes….” This should be described in more detail, as should the search for important regulatory regions tagged by non-exonic SNPs. They should also describe how the SNPs in Table 2 and Figs 2 and 3 were selected as “representative” of these genes. Presumably they chose the SNP with the strongest association (by p-value?) but this does not seem to be stated anywhere.

RESPONSE: We used the nearest gene approach to map SNP to a gene. The gene closest to a lead variant by genomic location was selected. We have revised the methods section and added the following on page 7, line 197:

“We used nearest gene approach to prioritize the potential causal genes. A gene with smallest genomic distance to a lead variant was selected”.

3. The data table in Fig. 3 describe a stronger association of rs581342 and neutropenia in COVID hospitalized persons of Hispanic genetic ancestry than in those of African ancestry (OR 1.65, p 8.84 x 10-6 ) that exceeds the authors’ defined significance threshold of p < 4.13 × 10-05, yet it is never mentioned. While the stronger OR is a point estimate based on a small number of cases (318 HIS vs. 1,788 AFR) and its confidence interval likely overlaps with that in AFR, the significance level merits a discussion despite its not being consonant with the authors’ hypothesis of a relationship to the known benign neutropenia among African Americans. Or is there an error in the table?

RESPONSE: We thank the Reviewer for this important point. We had initially focused discussing results based on our two largest populations AFR and EUR and neglected to mention other important results. We have now expanded discussion of *LMNA* and neutropenia in the Results section on page 11, line 341:

“…this association was also observed in HIS ancestry (OR_HIS_ = 1.65 [1.32 - 2.06], *P_HIS_* = 8.84 × 10^-06^) but was not in the larger EUR population.”

As well as the Discussion, on page 15, line 507:

“From this large-scale study across ancestries, we observed that a variant located in the LMNA gene locus was associated with a diagnosis of neutropenia in AFR ancestry and HIS, but not EUR which would otherwise would have been well powered to detect an association.  *LMNA* was associated with lower WBC counts across all ancestries, but its association with a lower fraction of neutrophils was observed in AFR and HIS only, but not EUR, in line with the overall association with diagnoses codes for neutropenia…”

4. The authors go into extensive discussions of PheWAS associations with idiopathic pulmonary fibrosis (lines 348-364), autoimmune diseases (lines 366-83) and neutropenia (lines 398-405) but provide almost no explanation of the observed lack of associations with pre-existing pulmonary disease aside from reiterating them in lines 319-20. The former three discussions could be cut back (particularly the seemingly tangential link to depression and anxiety in asthma in lines 360-61) and expand on reasons for the surprising lack of pulmonary disease associations or suggest research to clarify them.

RESPONSE: We appreciate the feedback and helpful suggestion. Please see edits throughout the revised discussion section streamlining the sections noted above. Additionally, we have added a more extensive discussion regarding the lack of association among risk factors such as COPD, starting on page 16, line 548:

“The PheWAS of risk alleles associated with severe COVID-19 did not observe an association between other chronic pulmonary conditions such as COPD, a risk factor for severe COVID manifestations.^2,3^ This absence of association allows us to discuss a few limitations of the PheWAS approach…”

5. Minor comments:

- Line 360: There may be a word missing in “…variations in this gene have also shown associations enhanced improvement….”

RESPONSE: As per the suggestion in the previous comment, we removed part of discussion section on depression and anxiety in asthma. This line was part of it and has been now removed.

- Table 1: numbers of Asian, Other, and Chronic Kidney Disease seem to be incorrectly punctuated.

RESPONSE: Errors corrected.

**References**

1.	Fang, H. *et al.* Harmonizing Genetic Ancestry and Self-identified Race/Ethnicity in Genome-wide Association Studies. *Am. J. Hum. Genet.* **105**, 763–772 (2019).

2.	Lee, S. C., Son, K. J., Han, C. H., Park, S. C. & Jung, J. Y. Impact of COPD on COVID-19 prognosis: A nationwide population-based study in South Korea. *Sci. Rep.* **11**, 3735 (2021).

3.	CDC. Coronavirus Disease 2019 (COVID-19) in the U.S. *Centers for Disease Control and Prevention* https://www.cdc.gov/coronavirus/2019-ncov/cases-updates/cases-in-us.html (2020).
